# 基于超高效液相色谱-静电场轨道阱高分辨质谱的深静脉血栓模型大鼠血浆代谢组学分析

**DOI:** 10.3724/SP.J.1123.2021.12024

**Published:** 2022-08-08

**Authors:** Yan GU, Peng ZANG, Jinxia LI, Yanyan YAN, Jia WANG

**Affiliations:** 1.山西大同大学医学院, 山西 大同 037009; 1. School of Medicine, Shanxi Datong University, Datong 037009, China; 2.大同市第三人民医院, 山西 大同 037000; 2. Datong No. 3 People’s Hospital, Datong 037000, China

**Keywords:** 超高效液相色谱-静电场轨道阱高分辨质谱, 代谢组学, 深静脉血栓, ultra-high performance liquid chromatography-electrostatic field orbitrap high resolution mass spectrometry (UHPLC-Orbitrap HRMS), metabolomics, deep vein thrombosis (DVT)

## Abstract

深静脉血栓(DVT)是一种血栓栓塞性疾病,具有高发病率、高死亡率和高后遗症3大特点。采用左股静脉不完全结扎加高渗盐水刺激建立DVT大鼠模型,使用超高效液相色谱-静电场轨道阱高分辨质谱(UHPLC-Orbitrap HRMS)检测假手术组与DVT模型组的血浆代谢谱,用主成分分析(PCA)及正交偏最小二乘-判别分析(OPLS-DA)对代谢组数据进行多元统计分析,观察两组间的代谢表型差异,将多变量模型分析中的变量重要性值(VIP>1)以及代谢物在模型组中的变化倍数(FC≤0.5或FC≥2,且*P*<0.05)作为差异代谢物筛选条件。最终在DVT模型组与假手术组间筛选得到27种差异代谢物,这些代谢物反映了DVT大鼠的代谢紊乱情况。具体表现为与假手术组相比,DVT模型组中三甲基胺氮氧化物(TMAO)、维生素K、鹅去氧胆酸、牛磺酸、1-甲基烟酰胺、7-酮胆固醇、反式十六烷基-2-烯醇肉碱、乙烯基乙酰甘氨酸、丙酰脯氨酸、咪唑乙酸、咪唑乙酸核糖苷、1,3,7-三甲基尿酸、1-丁胺、2-羟基异丙酸、吡哆醛、5*α*-四氢皮质酮、苯乳酸的水平显著升高;而3-脱氢肉碱、磷脂酰胆碱22∶6/20∶2(PC 22∶6/20∶2)、甘油二酯18∶3/20∶4(DG 18∶3/20∶4)、溶血磷脂酰胆碱20∶2(LysoPC 20∶2)、波维酸、鹅肌肽、L-肌肽、辛酸、羟基丙酮酸、3-羟基癸酸的水平显著降低。基于京都基因与基因组百科全书数据库(KEGG)代谢通路的差异丰度(DA)分析显示DVT模型大鼠与假手术组的代谢通路差异主要集中在初级胆汁酸生物合成、胆汁分泌、组氨酸代谢、亚油酸代谢、甘油磷脂代谢和*β*-丙氨酸代谢。紊乱的代谢物和代谢途径可为进一步深入理解DVT的病理机制、寻找诊断标志物及药物作用靶点提供参考。

## Abstract (en)

Deep vein thrombosis (DVT) is a venous thromboembolic disease characterized by high incidence, mortality, and sequelae. Therefore, the effective prevention of DVT has become a critical public health concern. However, due to its complexity, the pathophysiological mechanism of DVT remains unclear. Metabolomics can be employed to analyze disease characteristics and provide scientific evidence on the underlying mechanisms. In this study, an established left femoral vein ligation rat model of DVT (*n*=10) was used and compared with sham surgery controls (*n*=10). In the DVT group, rats were anesthetized using an intraperitoneal injection of 10% chloral hydrate (300 mg/kg), after which the hair was shaved and the groin disinfected. A 2-cm longitudinal incision was made along the midpoint of the left groin area, and then the left femoral vein was separated. The vein was partially ligated at its proximal end to shrink the blood vessel lumen to approximately half. Then, 0.4 mL of 10% hypertonic saline was slowly injected from the distal end of the left femoral vein. At the same time, the femoral vein turned dark red, which indicated the formation of thrombosis. Finally, the incision was sutured after verifying bleeding in the surrounding tissue. Keeping all other procedures the same as the DVT group, the vein in the control group was not ligated or stimulated using hyper-tonic saline. The abdominal aorta plasma from rats in each group was collected seven days later. Untargeted metabolomics analysis based on ultra-high performance liquid chromatography-electrostatic field orbitrap high resolution mass spectrometry (UHPLC-Orbitrap HRMS) was conducted to investigate the plasma metabolic profiles of the sham surgery control and DVT groups. Principal component analysis (PCA) and orthogonal to partial least squares discrimi-nant analysis (OPLS-DA) on metabolome data for multivariate statistical analysis were employed to assess differences in the metabolic profile between the two groups. The results revealed distinct profiles for the DVT and control groups. The selection criteria for the differential metabolites were the variable importance in the projection (VIP) values of OPLS-DA (VIP>1) and fold changes (FC) in the DVT group (FC≤0.5 or FC≥2, *P*<0.05). The resulting 27 differential metabolites reflecting a metabolic disorder in the DVT group were selected and analyzed. Of these, the levels of 17 metabolites significantly increased in the DVT group, including trimethylamine *N*-oxide (TMAO), 4-amino-2-methyl-1-naphthol, chenodeoxycholic acid, and 7-ketocholesterol, whereas the levels of 10 metabolites decreased, including 3-dehydroxycarnitine, phosphatidylcholine 22∶6/20∶2 (PC 22∶6/20∶2), diglyceride 18∶3/20∶4 (DG 18∶3/20∶4) and anserine. To identify the changes in the metabolic pathway reflected by these differential metabolites, a differential abundance (DA) analysis based on the Kyoto Encyclopedia of Genes and Genomes metabolic pathway was conducted. The results showed that the differences in the metabolic pathways between the DVT and control groups were mainly manifested in the primary bile acid biosynthesis, bile secretion, histidine metabolism, linoleic acid metabolism, glycerophospholipid metabolism, and *β*-alanine metabolism pathways. Among them, the primary bile acid biosynthesis and bile secretion pathways were upregulated in the DVT group, whereas the glycerophospholipid metabolism, linoleic acid metabolism, and *β*-alanine metabolism pathways were downregulated. The histidine metabolism pathway contained upregulated as well as downregulated metabolites, resulting in a DA score of 0. In conclusion, these results indicate that the plasma metabolic profiling of the DVT group was significantly altered, while the disordered metabolites and metabolic pathways could provide a reference to further understand the pathological mechanism of DVT and identify new drug targets.

深静脉血栓(DVT)是血栓栓塞性疾病的一种,是指血液在深静脉内不正常凝结引起的静脉回流障碍性疾病,多发生于下肢,深静脉血栓具有高发病率、高死亡率和高后遗症3大特点^[1]^。深静脉血栓患者在急性阶段若不能得到及时诊断和有效治疗,一些血栓可能会脱落,造成肺等重要脏器栓塞而死亡;而大约1/3的深静脉血栓患者会发生血栓后综合征,伴有肿胀、疼痛、皮肤改变和/或静脉溃疡,造成长期病痛,影响生活和工作。因此,有效预防深静脉血栓已经成为一个非常重要的公共卫生问题。然而,深静脉血栓是一种多因素疾病,涉及复杂的遗传、代谢和环境相互作用^[2]^,因此有必要深入了解深静脉血栓的病理生理特征,从而为该病的有效预防、早期诊断和药物作用靶点提供支撑。

代谢组学是系统生物学的重要组成部分,它以生物体内的小分子代谢产物为分析对象,研究疾病、外源性物质、生活方式、环境因素等对机体代谢组所产生的整体效应和系统作用,从而能够了解疾病的潜在分子机制,寻找具有诊断价值的生物标记物和/或治疗靶点^[3]^。代谢谱分析已成为研究复杂代谢疾病和实现精准医学的一种新方法。目前,采用代谢组学方法研究动物深静脉血栓代谢特征的报道较少。Sung等^[4]^建立了DVT小鼠模型并采用液相色谱-质谱联用技术和核磁共振氢谱(^1^H-NMR)技术,研究了DVT小鼠血清及血管壁的代谢特征,结果发现DVT的代谢紊乱主要表现为能量代谢、鞘脂和腺苷代谢。曹洁等^[5]^利用^1^H-NMR平台研究了DVT大鼠的尿液代谢物谱,发现差异代谢物主要与多个能量代谢途径有关,并可能成为DVT形成的候选生物标志物。代谢组学在血管疾病领域的应用提高了我们对DVT代谢改变的认识,并可以指导未来确定人类可能的DVT生物标志物。尽管在识别DVT差异代谢物及其在血栓形成中的改变方面取得了技术进步,但我们仍然不了解DVT代谢改变发生的机制和途径,因此仍需要进行大量的研究。

本研究采用基于超高效液相色谱-静电场轨道阱高分辨质谱(UHPLC-Orbitrap HRMS)的代谢组学技术测定了DVT大鼠的血浆代谢谱,采用多元及单元统计分析方法筛选与DVT相关的差异代谢物,分析代谢通路的变化,研究DVT形成的代谢机制,从而为进一步深入理解DVT的病理机制、寻找诊断标志物及药物作用靶点提供有价值的参考。

## 1 实验部分

### 1.1 仪器与试剂

LEGATO130微量注射泵(美国KD Scientific公司); WD-2102B酶标仪(北京六一生物科技有限公司); CX41显微镜(日本Olympus公司); 2235切片机(德国Leica公司); Heraeus Fresco17离心机、Vanquish超高效液相色谱、Q Exactive HFX高分辨质谱(美国Thermo Fisher公司)。

甲醇和乙腈均为色谱纯,购自德国CNW公司,色谱纯氨水购自美国Thermo Fisher公司,乙酸铵和内标(三甲胺-d_9_-*N*-氧化物、氯化胆碱-三甲基-d_9_、L-亮氨酸-5,5,5-d_3_、马尿酸-d_5_、邻氨基苯甲酸-环-^13^C_6_)均为色谱纯,购自美国Sigma公司。分别精密称取适量内标于100 mL容量瓶中,用甲醇-乙腈(1∶1, v/v)溶解至刻度,得到5种内标标准储备液,分别精密量取适量各内标标准储备液于同一100 mL容量瓶中,用甲醇-乙腈(1∶1, v/v)稀释至刻度,得到含有内标的提取液,其中各内标浓度如下:2 μmol/L(三甲胺-d_9_-*N*-氧化物)、0.06 μmol/L(氯化胆碱-三甲基-d_9_)、8 μmol/L(L-亮氨酸-5,5,5-d_3_)、0.6 μmol/L(马尿酸-d_5_)、0.04 μmol/L(邻氨基苯甲酸-环-^13^C_6_)。

### 1.2 实验动物与分组

20只SPF级SD大鼠,雄性,180~220 g,购自苏州西山生物技术有限公司,许可证号:SCXK(京)2019-0010。大鼠适应性饲养1周后,随机分为假手术组和DVT模型组,每组10只大鼠。

### 1.3 动物模型建立及样本采集

模型组参照文献^[6]^构建不完全结扎的大鼠股静脉血栓模型,即以10%水合氯醛腹腔注射麻醉(300 mg/kg),麻醉生效后,剃去大鼠腹股沟处鼠毛并消毒皮肤,沿左腹股沟区中点行2 cm纵向切口,分离左股静脉,在左股静脉近心端处绕以丝线不完全结扎,使血管腔缩小约1/2,以减慢血流,从左股静脉远心端缓慢注入10%高渗盐水0.4 mL,此时可观察到股静脉变为暗红色,提示血栓形成,检查周围组织无出血后缝合切口,外敷消毒纱布^[6]^。假手术组不结扎、不给予高渗盐水刺激,其余操作同模型组。造模后第7 d,各组大鼠用5%水合氯醛腹腔麻醉,腹主动脉取血,一部分血液置于冷水浴冷却的肝素抗凝管中,用酶联免疫吸附试验(ELISA)检测血中D-二聚体水平;另一部分血液同样置于冷水浴冷却的肝素抗凝管中,混匀,以3000 r/min的转速于4 ℃离心10 min,分离血浆并保存于-80 ℃冰箱中用于代谢谱分析。同时经原切口取出各组大鼠股静脉血栓段,进行常规固定、包埋、切片,行苏木精-伊红染色法后置于光镜下观察两组大鼠局部血栓形成静脉段组织形态学的差异。

### 1.4 样本制备

取血浆100 μL,加入400 μL含内标的提取液甲醇-乙腈(1∶1, v/v),涡旋混匀30 s,冰水浴超声10 min, -40 ℃静置1 h以沉淀蛋白质,以12000 r/min的转速于4 ℃离心15 min,取上清液进样分析。同时所有样品取等量上清液混合制备质量控制(QC)样品。

### 1.5 分析条件

色谱柱:Waters UPLC BEH Amide色谱柱(100 mm×2.1 mm, 1.7 μm);流动相:A相为水,含25 mmol/L乙酸铵和25 mmol/L氨水,B相为乙腈;柱温:35 ℃;流速:0.5 mL/min;自动进样器温度:4 ℃;进样量:2 μL。梯度洗脱程序:0~0.5 min, 95%B; 0.5~7.0 min, 95%B~65%B; 7.0~8.0 min, 65%B~40%B; 8.0~9.0 min, 40%B; 9.0~9.1 min, 40%B~95%B; 9.1~12.0 min, 95%B。

离子源:电喷雾电离(ESI)源,正负离子切换模式;鞘层气体流速:30 Arb;辅助气体流速:25 Arb;毛细管温度:350 ℃;采集方法:信息依赖型采集(IDA)模式;采集时间:12 min;扫描范围:*m/z* 70~1050;全MS分辨率:60000, MS/MS分辨率:7500;归一化碰撞能量:10%、30%、60%;喷射电压:3600 V(ESI^+^)/-3200 V(ESI^-^)。

### 1.6 数据分析

原始数据经ProteoWizard软件转成mzXML格式后,使用XCMS程序进行峰提取、峰对齐、积分等处理^[7]^,得到原始离子峰表。删除组内缺失值>50%的离子峰,同时用每个离子峰最小值的1/2填补缺失值,并采用内标进行校正,最后与BiotreeDB(V2.1)二级质谱数据库匹配进行代谢物注释。多元统计分析包括主成分分析(PCA)及正交偏最小二乘法-判别分析(OPLS-DA),由SIMCA-P软件(16.0.2版本,Umetrics AB公司,瑞典)完成。OPLS-DA模型的可靠性和预测性采用7次循环交互验证法评价,参数*R*^2^*Y*、*Q*^2^(*R*^2^*Y*表示模型对*Y*变量的解释率,*Q*^2^表示模型预测能力)用以评估模型质量;响应排序检验(*n*=200)用来考察OPLS-DA模型的准确性,即将原始分类的*Y*矩阵、200次不同排列的*Y*矩阵与*R*^2^*Y*、*Q*^2^进行线性回归,得到的回归直线与*y*轴的截距值分别为*R*^2^和*Q*^2^,用以判断模型是否过拟合。变量重要性(VIP)值用来评价OPLS-DA模型中每个变量的贡献。Student *t*检验用来检验代谢物在两组间差异的显著性,*P*<0.05认为具有统计学差异。人类代谢组数据库(HMDB)用来确定差异代谢物的类别,差异代谢物的代谢通路分析主要基于京都基因与基因组百科全书数据库(KEGG)。

## 2 结果与讨论

### 2.1 两组大鼠行为学比较

造模前两组大鼠均精神状况良好,四肢活动及饮食正常。造模后假手术组大鼠精神状态仍良好,左下肢无明显肿胀,行动无明显受限。模型组大鼠造模后精神状况一般,左下肢明显肿胀,颜色青紫,活动减少。

### 2.2 两组大鼠血液中D-二聚体水平比较

D-二聚体是交联纤维蛋白的特异性降解产物之一,是继发性纤溶的特有代谢产物。D-二聚体的水平与血栓的大小和活动性相关,DVT形成的同时纤溶系统也被激活,血液中D-二聚体浓度上升^[8]^,因而D-二聚体是临床中DVT的常用辅助诊断指标。通过ELISA检测血中D-二聚体水平,结果发现假手术组及DVT模型组中D-二聚体的平均质量浓度分别为(41.67±4.57) μg/L和(108.77±9.44) μg/L,模型组的数值显著升高,差异具有统计学意义(*P*=1.1×10^-28^)。

### 2.3 两组大鼠静脉血栓段的组织形态学差异

如图1所示,假手术组中整个血管内膜表面平整,内皮细胞排列整齐,少量内皮细胞脱落,管腔中无血栓形成(见图1a);模型组中静脉管腔被血栓堵塞,血管内皮剥落,血管壁炎性细胞浸润较为严重(见图1b)。综上所述,显示大鼠深静脉血栓模型构建成功。

**图1:**
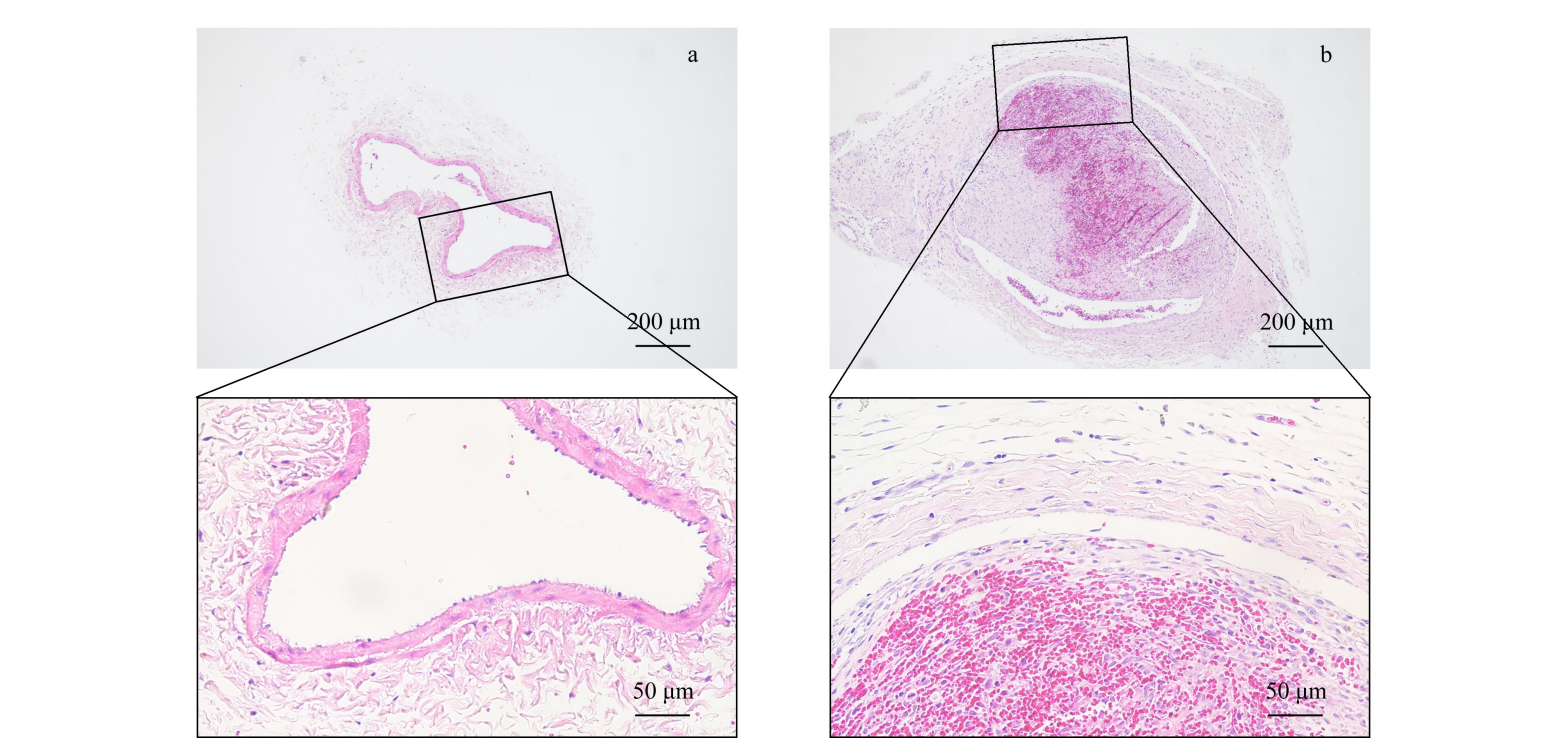
(a)假手术组与(b)DVT模型组大鼠静脉血栓的苏木精-伊红染色

### 2.4 数据质量控制

为了了解分析平台的稳定性,首先将QC样品、假手术组和DVT模型组提取得到的离子峰进行PCA分析,如图2所示,正、负离子模式下QC样本紧密聚集在一起。其次计算QC样品中内标峰面积的相对标准偏差,结果发现正离子模式下3个内标三甲胺-d_9_-*N*-氧化物、氯化胆碱-三甲基-d_9_和L-亮氨酸-5,5,5-d_3_峰面积的相对标准偏差分别为1.35%、3.13%和5.64%,负离子模式下内标L-亮氨酸-5,5,5-d_3_、马尿酸-d_5_和邻氨基苯甲酸-环-^13^C_6_峰面积的相对标准偏差分别为10.8%、4.23%和5.16%,所有内标峰面积的相对标准偏差均小于15%。以上结果说明数据质量较高,分析平台具有良好的稳定性。

**图2:**
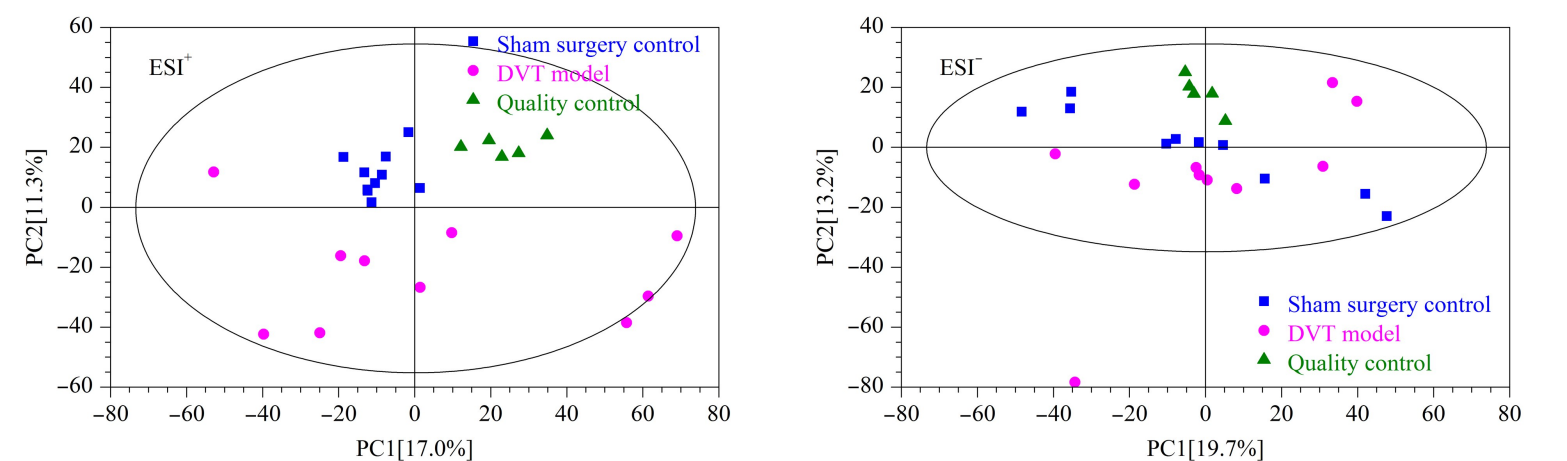
ESI模式下血浆样本的PCA得分图

### 2.5 DVT大鼠血浆代谢物谱的改变

采用UHPLC-Orbitrap HRMS平台在正、负离子模式下分别采集假手术组和DVT模型组的血浆代谢物谱信息,数据经过预处理后正、负离子模式可分别得到4292和3501个变量。计算模型组中所有变量相对于假手术组的变化倍数(FC),并采用火山图直观观察DVT模型组中所有变量的变化情况,火山图中偏离轴线的点表示在模型组中发生了明显改变的变量(*P*<0.05)(见图3)。同时采用多元统计分析,通过建立可靠的数学模型来进一步研究DVT大鼠的代谢谱特点。首先进行PCA分析,由图4a可见,DVT模型组与假手术组有分离的趋势。为了进一步放大组间差异,将假手术组和模型组的血浆代谢谱数据进行OPLS-DA分析,这种方法可以过滤掉代谢物中与分类变量不相关的正交变量,并对非正交变量和正交变量分别分析,从而获得更加可靠的代谢物组间差异^[9]^。

**图3:**
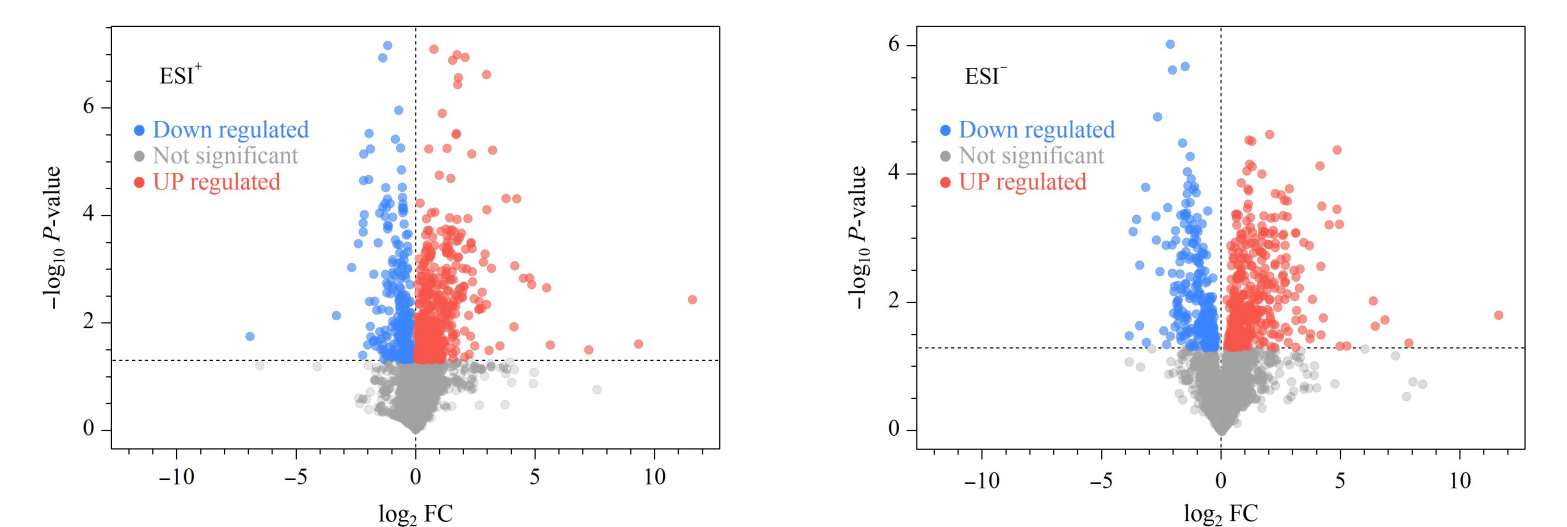
假手术组与DVT模型组大鼠血浆代谢物火山图

**图4:**
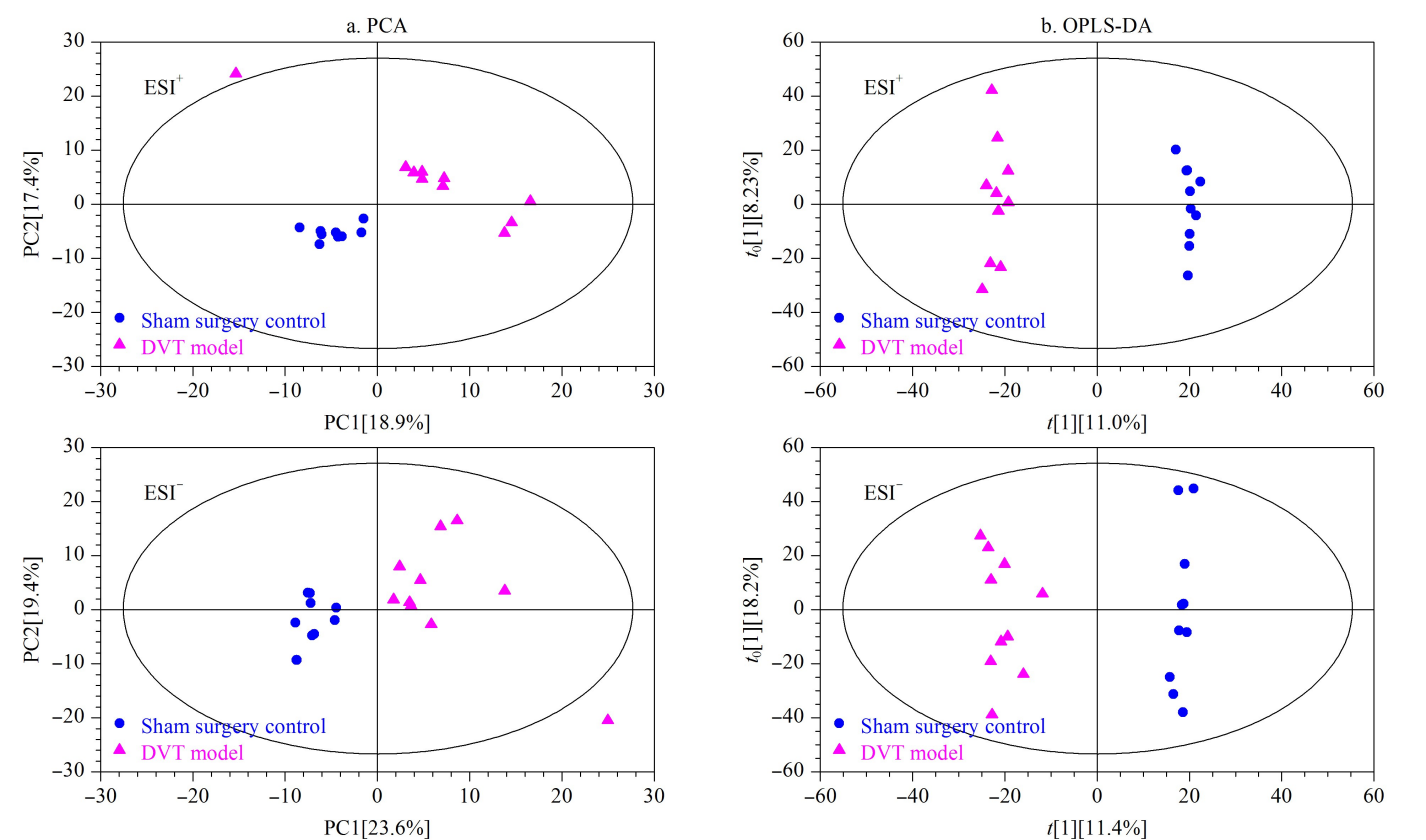
假手术组与DVT模型组大鼠血浆代谢物的(a)PCA和(b)OPLS-DA得分图

由图4b可见,两组样本区分非常显著,说明DVT大鼠血浆代谢物谱较假手术组发生了明显改变。经7次循环交互验证得到的模型评价参数(*R*^2^*Y*、*Q*^2^)分别为0.995、0.738(正离子模式)和0.978、0.721(负离子模式), *R*^2^*Y*、*Q*^2^均大于0.5,说明建立的OPLS-DA模型稳定可靠。响应置换检验的*R*^2^、*Q*^2^截距分别为0.23、-0.17(正离子模式)和0.27、-0.19(负离子模式),表明模型有效,不存在过拟合现象。

### 2.6 差异代谢物筛选及鉴别

筛选在两个OPLS-DA模型中VIP>1,且FC≤0.5或FC≥2,同时*P*<0.05的变量,经与BiotreeDB(V2.1)二级质谱数据库匹配后,最终在正、负离子模式下共筛选得到27种差异代谢物,见表1。这些代谢物在假手术组与DVT模型组间存在着显著性差异,反映了DVT大鼠的代谢紊乱情况。图5可视化地展示了差异代谢物在假手术组和DVT模型组间的变化情况。具体表现为,与假手术组相比,DVT模型组中三甲基胺氮氧化物(TMAO)、维生素K、鹅去氧胆酸、牛磺酸、1-甲基烟酰胺、7-酮胆固醇、反式十六烷基-2-烯醇肉碱、乙烯基乙酰甘氨酸、丙酰脯氨酸、咪唑乙酸、咪唑乙酸核糖苷、1,3,7-三甲基尿酸、1-丁胺、2-羟基异丙酸、吡哆醛、5*α*-四氢皮质酮、苯乳酸的水平显著升高;而3-脱氢肉碱、磷脂酰胆碱22∶6/20∶2(PC 22∶6/20∶2)、甘油二酯18∶3/20∶4(DG 18∶3/20∶4)、溶血磷脂酰胆碱20∶2(LysoPC 20∶2)、波维酸、鹅肌肽、L-肌肽、辛酸、羟基丙酮酸、3-羟基癸酸的水平显著降低。

**图5:**
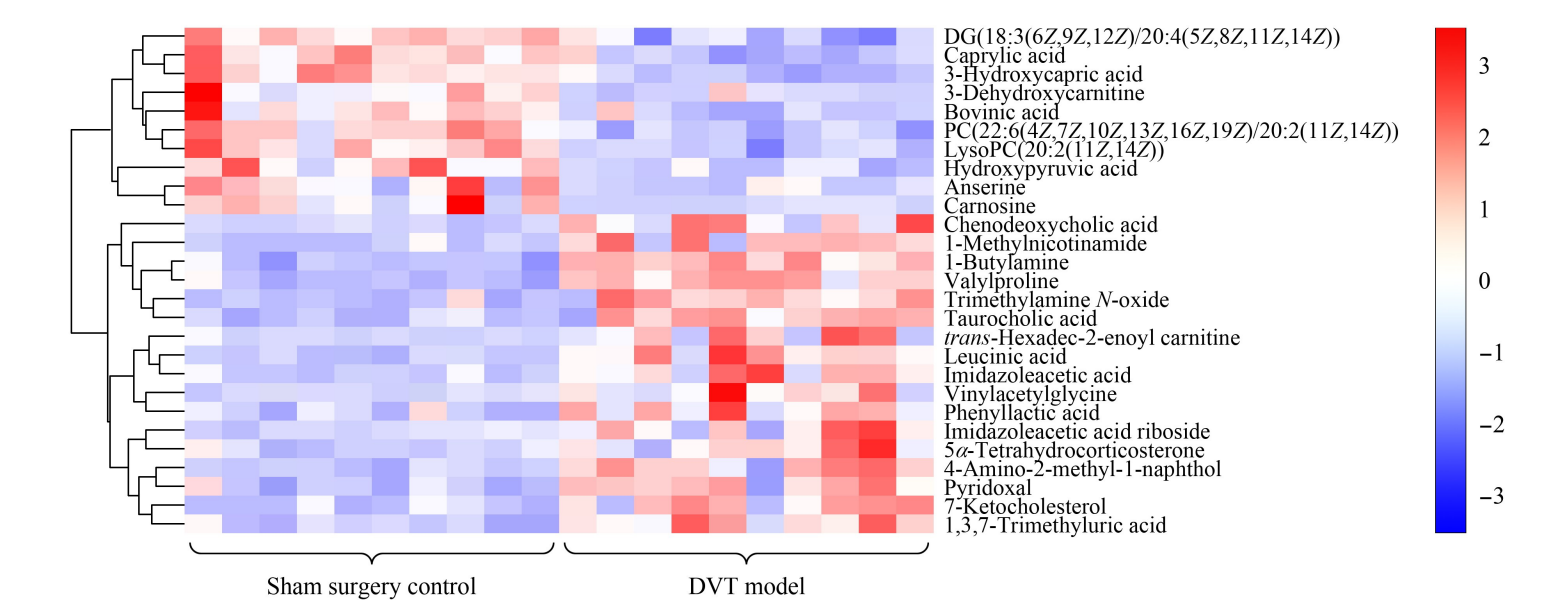
假手术组与DVT模型组大鼠血浆差异代谢物热图

**表1:** 假手术组与DVT模型组间的血浆差异代谢物

Metabolite	FC	P-value	ESI^±^	Category
Trimethylamine N-oxide	2.82	2.4×10^-4^	+	aminoxides
4-Amino-2-methyl-1-naphthol	2.86	1.0×10^-3^	+	naphthols
Chenodeoxycholic acid	3.20	1.6×10^-2^	+	bile acids
Taurocholic acid	3.15	7.8×10^-4^	-	bile acids
7-Ketocholesterol	2.40	2.0×10^-3^	+	cholesterols
trans-Hexadec-2-enoyl carnitine	3.05	2.6×10^-2^	+	fatty acids
Leucinic acid	6.02	1.0×10^-3^	-	branched fatty acids
1-Methylnicotinamide	3.91	1.0×10^-3^	+	pyridinecarboxylic acids
Vinylacetylglycine	2.85	2.7×10^-2^	+	amino acids
Valylproline	3.40	1.1×10^-6^	+	amino acids
1-Butylamine	3.02	5.7×10^-8^	+	amines
Imidazoleacetic acid riboside	2.13	2.6×10^-2^	+	nucleosides
1,3,7-Trimethyluric acid	3.94	1.0×10^-3^	+	purines
5α-Tetrahydrocorticosterone	2.80	1.7×10^-2^	-	steroids
Phenyllactic acid	2.50	2.0×10^-3^	-	phenylpropanoids
Pyridoxal	2.55	4.1×10^-4^	+	pyridines
Imidazoleacetic acid	2.08	4.0×10^-3^	-	organic heterocycles
3-Dehydroxycarnitine	0.36	3.8×10^-2^	+	keto acids
PC(22∶6(4Z,7Z,10Z,13Z,16Z,19Z)/20∶2(11Z,14Z))	0.48	2.9×10^-5^	+	glycerophospholipids
DG(18∶3(6Z,9Z,12Z)/20∶4(5Z,8Z,11Z,14Z))	0.41	6.3×10^-5^	+	glycerolipids
LysoPC(20∶2(11Z,14Z))	0.44	2.1×10^-4^	+	glycerophospholipids
Anserine	0.36	1.9×10^-2^	-	peptidomimetics
L-Carnosine	0.10	2.2×10^-2^	-	peptidomimetics
Caprylic acid	0.43	6.8×10^-5^	-	fatty acids
Bovinic acid	0.49	5.0×10^-3^	-	fatty acids
3-Hydroxycapric acid	0.36	1.7×10^-5^	-	fatty acids
Hydroxypyruvic acid	0.36	4.0×10^-3^	-	organic acids

PC: phosphatidylcholine; DG: diglyceride.

### 2.7 代谢通路分析

为了寻找差异代谢物体现的代谢通路的改变,采用了基于KEGG代谢通路的差异丰度(DA)分析,结果见图6。由图可知,DVT模型大鼠与假手术组的代谢通路差异主要集中在组氨酸代谢、胆汁分泌、亚油酸代谢、甘油磷脂代谢、初级胆汁酸生物合成和*β*-丙氨酸代谢,且每一代谢通路中至少包含2个差异代谢物。其中,胆汁酸分泌通路(涉及上调的1-甲基烟酰胺、鹅去氧胆酸和牛磺酸)和初级胆汁酸生物合成通路(涉及上调的鹅去氧胆酸和牛磺酸)在DVT模型组中均上调;甘油磷脂代谢通路(涉及下调的磷脂酰胆碱、溶血磷脂酰胆碱)、亚油酸代谢通路(涉及下调的磷脂酰胆碱、溶血磷脂酰胆碱、波维酸)、*β*-丙氨酸代谢通路(涉及下调的鹅肌肽和L-肌肽)在DVT模型组中均下调;组氨酸代谢通路中既包含上调的差异代谢物咪唑乙酸核糖苷、咪唑乙酸,也包含下调的鹅肌肽和L-肌肽,因而其差异丰度得分为0。

**图6:**
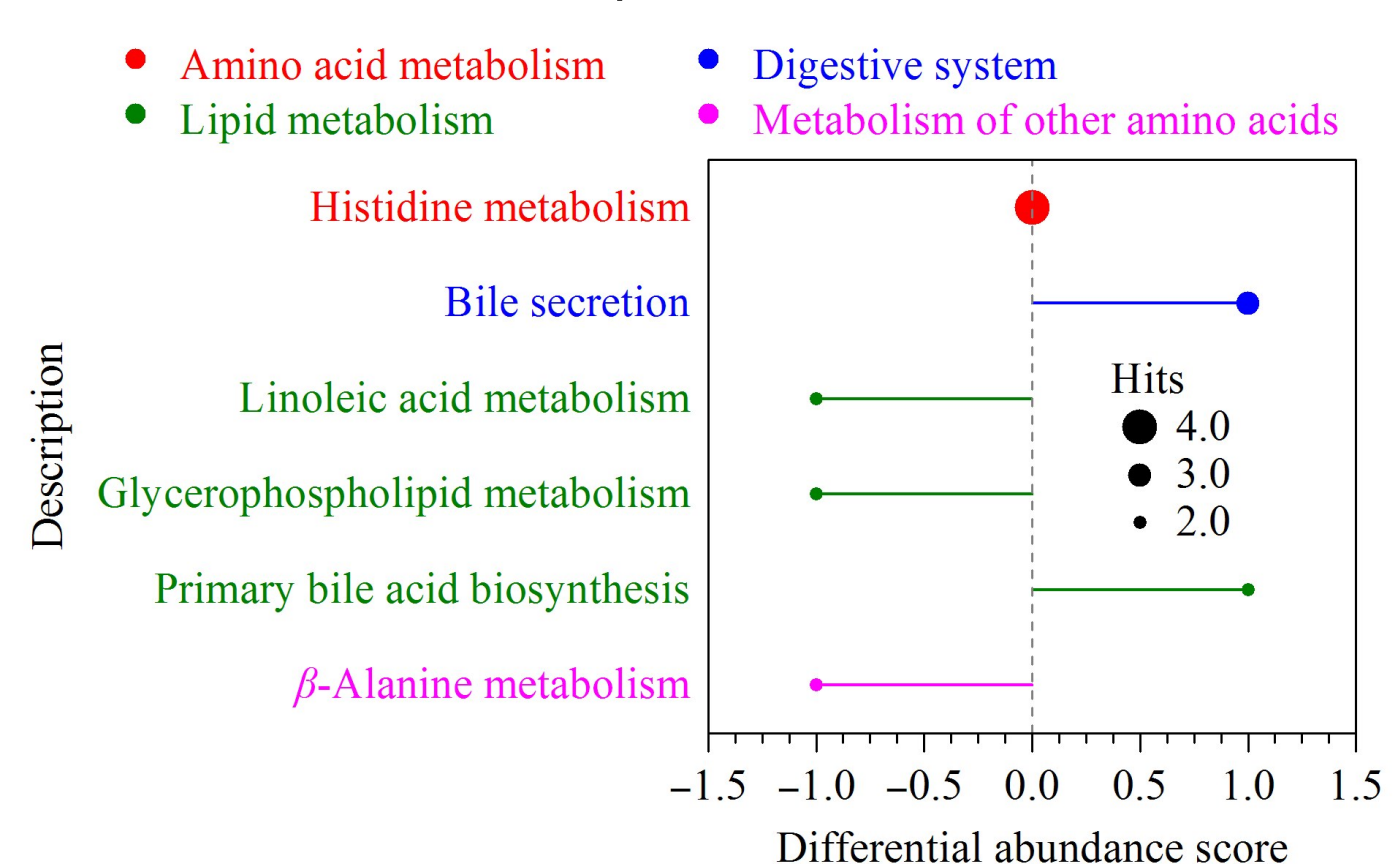
基于京都基因与基因组百科全书数据库的通路差异丰度得分图

在本研究中,采用代谢组学方法观察到DVT模型大鼠的血浆代谢表型与假手术组相比存在明显差异,多种代谢产物在DVT模型组中发生了显著改变(FC≤0.5或FC≥2),代谢通路差异主要集中在初级胆汁酸生物合成、胆汁分泌、组氨酸代谢、亚油酸代谢、甘油磷脂代谢和*β*-丙氨酸代谢。

胆汁酸及脂代谢:与假手术组比较,鹅去氧胆酸、牛磺酸、1-甲基烟酰胺水平在DVT模型组中均显著升高。鹅去氧胆酸和牛磺酸是胆固醇在肝脏转化生成的初级胆汁酸,1-甲基烟酰胺是胆汁酸分泌途径中的代谢物,它们的水平升高可能提示DVT大鼠初级胆汁酸生物合成以及胆汁酸分泌途径被激活。研究证实胆汁酸是激活多种细胞内信号通路的信号分子^[10]^,血浆胆汁酸水平增多,能够激活法尼类X受体(FXR)^[11]^, FXR在调节肝内胆汁酸合成和分泌以及脂质和葡萄糖代谢中起着关键作用^[12]^,而鹅去氧胆酸、胆酸及其结合物牛磺酸是FXR的有效内源性配体^[13]^,动物实验证实它们通过激活FXR可显著降低空腹血糖、血浆胆固醇、甘油三酯、脂肪酸等所有脂质标记物^[14]^。在DVT模型组中,我们发现除了变化倍数较大的PC(22∶6/20∶2)、DG(18∶3/20∶4)、LysoPC(20∶2)、辛酸、脂肪酸*β*-氧化的中间产物3-羟基癸酸、亚油酸的共轭代谢物波维酸浓度显著下降外,葡萄糖和其他多种脂类代谢物浓度也在模型组中显著下降(*P*<0.05,但FC没有达到差异代谢物筛选条件),如胆固醇、PC(18∶1/16∶0)、PC(22∶6/20∶1)、PC(22∶5/18∶2)、PC(22∶4/15∶0)、PC(18∶2/15∶0)、LysoPC(20∶4)、DG(18∶3/20∶3)、LysoPC(20∶2)和花生四烯酸。因而,本实验中葡萄糖及脂类代谢产物浓度在DVT模型组中显著下调也同样证实了升高的初级胆汁酸对糖脂代谢的调节作用。

组氨酸代谢途径中有4条代谢去路,最终分别代谢为鹅肌肽、L-天冬氨酸、L-谷氨酸和硫脲酸。实验发现组氨酸向鹅肌肽代谢去路中的代谢产物L-肌肽和鹅肌肽水平在模型组中均下调,说明这条代谢途径不是DVT大鼠体内组氨酸代谢的主要途径。然而,我们发现组氨酸向L-天冬氨酸代谢去路中的2个中间产物咪唑乙酸核糖苷、咪唑乙酸在模型组中均上调,说明DVT大鼠体内组氨酸代谢可能主要通过向L-天冬氨酸转变的途径完成。

*β*-丙氨酸代谢途径中涉及的差异代谢物鹅肌肽和L-肌肽在模型组中显著下调。L-肌肽和鹅肌肽均是二肽,它们都可以分解成*β*-丙氨酸,模型组中我们发现*β*-丙氨酸浓度显著增多(*P*=4.0×10^-3^, FC=1.7),说明DVT大鼠体内鹅肌肽和L-肌肽的分解代谢加强,从而生成更多的*β*-丙氨酸。有文献报道,鹅肌肽和L-肌肽在抗氧化和抗炎反应中具有重要的生理作用^[15,16]^,而DVT模型组中这两种物质的下调,可能使它们不能发挥对静脉血栓形成后炎症反应的调节作用,当然这种推测还需要进一步的实验去验证。

其他差异代谢物:TMAO是三甲胺的氧化产物,是动物和人类常见的代谢物。TMAO主要在肠道菌群的作用下,由食物中的磷脂酰胆碱或肉碱转化而成。研究报道血液中TMAO浓度升高会增强血小板的反应性、促进血栓的形成^[17,18]^,本实验发现模型组中TMAO水平较假手术组显著上调,也进一步印证了TMAO与血栓形成的关联性。维生素K浓度在模型组中明显升高。维生素K是*γ*-羧化酶的辅酶,主要参与肝合成凝血因子,具有促进凝血的作用,模型组中维生素K水平增加说明模型组大鼠体内处于高凝状态,易于血栓的形成。羟基丙酮酸,存在于从细菌到人类的所有生物体中。羟基丙酮酸主要在丝氨酸转移酶催化下由L-丝氨酸转变而来,随后一方面通过生成甘油酸或2-羟基-3-氧丙酸酯进入抗坏血酸和醛酸代谢途径,另一方面通过生成羟基乙醛进入维生素B6代谢途径,并与2-氧代丁酸、丙氨酸反应后最终转变成吡哆醇和吡哆醛。我们发现DVT模型组中羟基丙酮酸浓度明显下降,而吡哆醛浓度明显升高,说明DVT大鼠体内羟基丙酮酸主要向生成维生素B6的途径转化。与假手术组相比,DVT大鼠的肉碱代谢也发生了明显改变,表现为3-脱氢肉碱显著减少,反式十六烷基-2-烯酰基肉碱显著增多。3-脱氢肉碱是L-肉碱在肠道细菌作用下分解生成的中间产物^[19]^, 3-脱氢肉碱进一步分解为三甲胺,后者随即转化为TMAO^[20]^。由于本实验发现TMAO在DVT模型组中显著升高,因此我们推测DVT大鼠的肠道菌群较为活跃,提高了L-肉碱的肠道代谢能力。反式十六烷基-2-烯酰基肉碱是一种特殊的酰基肉碱,其在体内的积累能够促进机体促炎症因子的表达,并诱导c-Jun氨基末端激酶(JNK)和细胞外信号调节激酶(ERK)的磷酸化^[21]^。静脉血栓形成后血管壁通常会伴有炎症细胞的浸润,本实验中反式十六烷基-2-烯酰基肉碱在DVT大鼠血浆中显著增多可能是静脉血栓形成后发生血管壁炎症反应的原因之一。此外,实验还发现在DVT大鼠血浆中7-酮胆固醇水平显著升高。7-酮胆固醇是一种氧化甾醇,是胆固醇的主要氧化产物,正常情况下在血液循环中的含量较低,但在一些心血管疾病中7-酮胆固醇会在血浆和组织中积累并与疾病的发病机制相关^[22]^。研究发现,7-酮胆固醇能诱导血管内皮细胞促炎细胞因子白细胞介素-1*β*(IL-1*β*)、IL-6、IL-8、肿瘤坏死因子-*α*(TNF-*α*)和环氧合酶-2(COX2)的mRNA表达以及提高COX2酶的活性,从而引起内皮细胞的炎症反应^[22]^; 7-酮胆固醇损伤内皮细胞并增加凝血蛋白的表达和释放^[23]^; 7-酮胆固醇诱导细胞内活性氧(ROS)的生成,激活内皮细胞中与炎症反应和细胞凋亡有关的信号通路^[24]^。这些研究表明7-酮胆固醇对机体的毒性作用主要与其损害血管内皮细胞的功能有关,内皮细胞功能受损会引起动脉粥样硬化、血栓形成等心血管疾病^[24]^。本实验DVT模型组中7-酮胆固醇浓度较假手术组明显增高,提示7-酮胆固醇可能通过损伤DVT大鼠的血管内皮细胞、诱导炎症反应等作用促进血栓的形成。

## 3 结论

本研究采用基于UHPLC-Orbitrap HRMS技术的代谢组学方法研究了DVT大鼠的血浆代谢特征。结果表明,DVT大鼠的血浆代谢谱与假手术组相比发生了显著变化,主要体现在初级胆汁酸生物合成、胆汁分泌、组氨酸代谢、亚油酸代谢、甘油磷脂代谢和*β*-丙氨酸代谢方面发生了不同程度的紊乱。此外,实验还发现DVT模型组中一些与血栓形成和炎症相关的代谢物如TMAO、维生素K、反式十六烷基-2-烯酰基肉碱、7-酮胆固醇、鹅肌肽和L-肌肽的水平同样发生了显著改变。本文的研究结果可为进一步深入探讨深静脉血栓的病理代谢过程、寻找诊断标志物以及药物作用靶点提供参考,也表明代谢组学方法是研究疾病机理的一种重要手段。
